# Bladder pseudo-tumor: case report of vesical tamm-horsfall protein deposit

**DOI:** 10.1590/S1677-5538.IBJU.2019.0522

**Published:** 2020-02-20

**Authors:** Marcelo Langer Wroclawski, Willy Roberto Camargo Baccaglini, Cristiano Linck Pazeto, Luisa Emanuela Biseo Henriques, Alexandre Kiyoshi Hidaka, Felipe Ko Chen, Milton Borreli, Renne Zon Filippi

**Affiliations:** 1 Hospital Israelita Albert Einstein São PauloSP Brasil Hospital Israelita Albert Einstein, São Paulo, SP, Brasil; 2 Faculdade de Medicina do ABC Disciplina de Urologia Santo AndréSP Brasil Disciplina de Urologia, Faculdade de Medicina do ABC, Santo André, SP, Brasil

## INTRODUCTION

Urothelial carcinomas (UC) are malignant tumors that correspond to more than 90% of the bladder tumors (1). The main sign of UC is hematuria, however with the routine use of imaging exams, more patients are being diagnosed whilst asymptomatic. On ultrasonography (US), UCs present as a focal bladder wall thickening and/or a polypoid lesion (2).

Nevertheless, these findings may be due to several other malignant and non-malignant differential diagnoses, such as nephrogenic adenoma, inverted papilloma, leiomyoma, amyloidosis, glandular cystitis, endometriosis, bladder xanthoma, among others (3–6). Cystoscopy is the gold standard procedure to investigate patients with suspicion of any bladder neoplasia.

Our objective is to report a case of Tamm-Hosrsfall protein deposit in the bladder wall, mimicking a vesical UC.

## CASE REPORT

**Figure 1:**
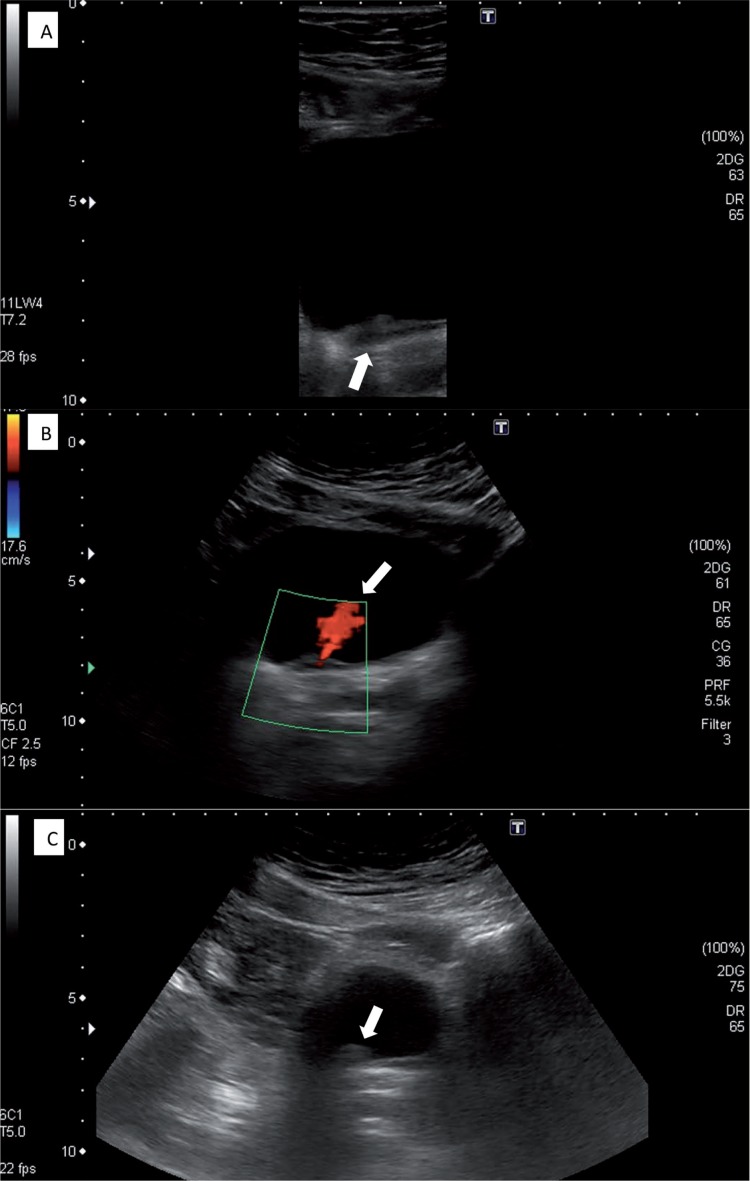
A: US with an area of focal thickening in the bladder floor (arrow); B: The focal thickening in the bladder floor is close to the ureteral meatus (arrow show the ureteric jet in US doppler); C: Nodular lesion in the bladder floor, close to the focal thickening (arrow).

A 51-year-old asymptomatic man, with no history of hematuria, underwent to a routine US. The exam demonstrated a bladder with regular walls, except for an area of focal thickening and a nodular lesion in the bladder floor, close to the right ureteral meatus (Figure-1). Serum and urinary laboratory tests were normal.

**Figure 2:**
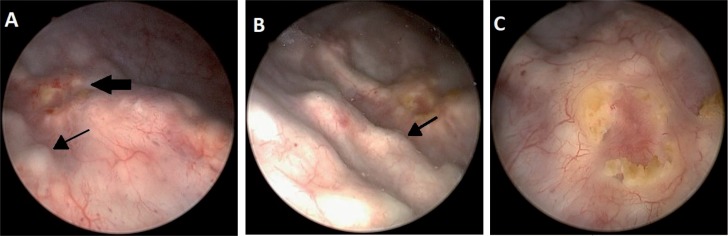
(cystoscopy) – A) right peri-meatal region, which is evidencing ulcerated lesion (thick arrow) and lesions elevated with intact mucosa (narrow arrow); B) Image focused on elevated lesions (thick arrow); C) image focused on ulcerated lesion.

**Figure 3:**
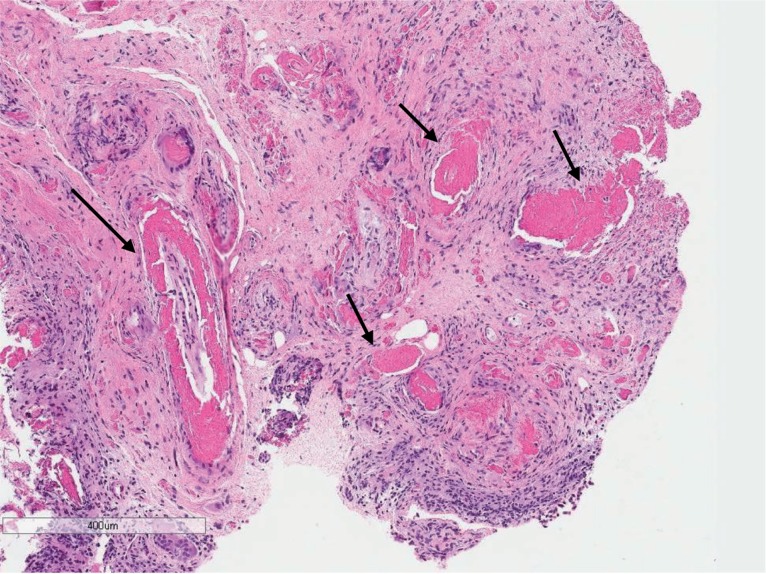
Hematoxylin and Eosin (H&E) stain - Bladder biopsy: deposits of eosinofilic material in the lamina propria (arrows).

**Figure 4:**
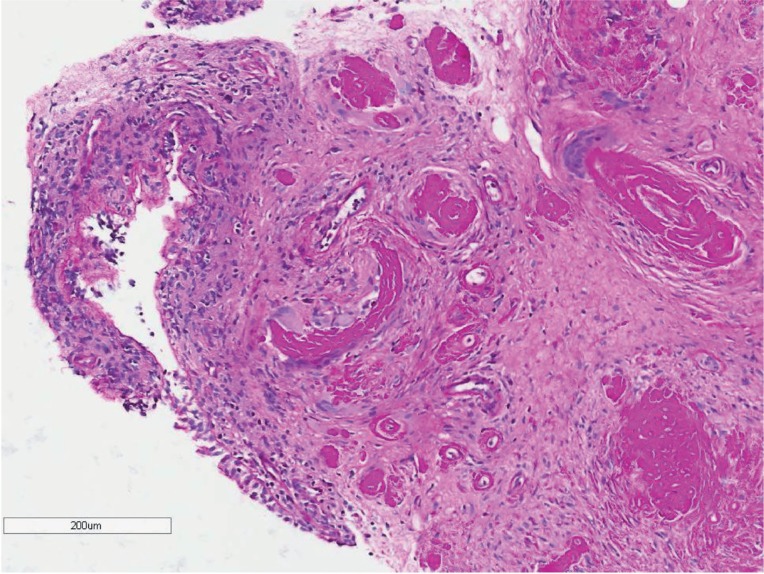
Periodic Acid Schif (PAS) stain: eosinofilic material deposits strongly positive by the PAS stain.

Cystoscopy found three elevated lesions in the right lateral vesical wall, each one with about 0.5cm, all of which with intact mucosa. Additionally, there was an ipsilateral ulcerated peri-meatal lesion (Figure-2). All lesions were cold-cup biopsied and the pathological analysis revealed deposition of an eosinophilic proteinaceous substance throughout the mucosa and around the vessels. This was also associated with a mixed inflammatory process at the lamina propria, without evidence of cellular atypia (Figures 3 and 4). The search for infectious agents and amyloid protein (red-congo) were negative. The findings led to the diagnosis of Tamm-Horsfall protein deposition (THP). The patient remained asymptomatic and had no complications following the procedure.

## DISCUSSION

The THP is a high molecular weight glycoprotein synthesized in the ascending portion of the Henle loop, and in the distal convoluted tubule. THP is abundant in normal human urine. Its actual physiological function remains unknown, but there is a hypothesis about a possible protective factor against urinary tract infections, lithogenesis, and some nephropathies (7–9).

The etiology for THP deposit is still unclear, however it is most likely related to mucosal changes, such as inflammation and necrosis (7–9).

A series of three patients with atypical THP mimichking tumor at the peri-pelvic and peri-renal fat tissues has been reported. In addition to the initial bladder carcinoma diagnostic hypothesis, renal pelvic neoplasia and urinary tuberculosis were also suspected (10). Another report presented a patient with a ureteral lesion associated with hydronephrosis, which suggested a tumor, but exactly like our case, histology favored THP deposition (11).

A large study consisting of 247 bladder biopsies and 15 specimens of cystectomy identified the presence of THP deposition in the bladder tissue in 18 cases (6.9%). The cystectomy cases presented positive biopsies for THP deposition in 60% of the patients, higher than isolated biopsies (3.6%). The author describes a typical pathological finding characterized by whitish masses with discrete eosinophilic deposition (12). However, our patient, beyond the THP deposits mimicking a bladder tumor, did not present any other bladder pathology or symptoms.

There are reports that have identified association between bladder wall THP deposition and interstitial cystitis (13, 14). Additionally, patients with interstitial cystitis have been reported to have changes in THP when compared to control groups.

Our case demonstrates that THP deposition in the bladder may be one of the differential diagnoses for bladder lesions, mainly when the lesion does not have the usual papillary aspect and appears to be in a sub-urothelial layer.
